# Acceptability and Usability of a Wearable Device for Sleep Health Among English- and Spanish-Speaking Patients in a Safety Net Clinic: Qualitative Analysis

**DOI:** 10.2196/43067

**Published:** 2023-06-05

**Authors:** Larissa Purnell, Maribel Sierra, Sarah Lisker, Melissa S Lim, Emma Bailey, Urmimala Sarkar, Courtney R Lyles, Kim H Nguyen

**Affiliations:** 1 School of Public Health University of California Berkeley Berkeley, CA United States; 2 Division of General Internal Medicine School of Medicine University of California San Francisco San Francisco, CA United States; 3 Center for Vulnerable Populations School of Medicine University of California San Francisco San Francisco, CA United States; 4 Redwood Pulmonary Medical Associates Redwood City, CA United States; 5 Somnology Redwood City, CA United States; 6 Department of Epidemiology and Biostatistics School of Medicine University of California San Francisco San Francisco, CA United States

**Keywords:** health equity, medical informatics, sleep disorders, user-centered design, wearable electronic devices

## Abstract

**Background:**

Sleep disorders are common and disproportionately affect marginalized populations. Technology, such as wearable devices, holds the potential to improve sleep quality and reduce sleep disparities, but most devices have not been designed or tested with racially, ethnically, and socioeconomically diverse patients. Inclusion and engagement of diverse patients throughout digital health development and implementation are critical to achieving health equity.

**Objective:**

This study aims to evaluate the usability and acceptability of a wearable sleep monitoring device—SomnoRing—and its accompanying mobile app among patients treated in a safety net clinic.

**Methods:**

The study team recruited English- and Spanish-speaking patients from a mid-sized pulmonary and sleep medicine practice serving publicly insured patients. Eligibility criteria included initial evaluation of obstructed sleep apnea, which is most appropriate for limited cardiopulmonary testing. Patients with primary insomnia or other suspected sleep disorders were not included. Patients tested the SomnoRing over a 7-night period and participated in a 1-hour semistructured web-based qualitative interview covering perceptions of the device, motivators and barriers to use, and general experiences with digital health tools. The study team used inductive or deductive processes to code interview transcripts, guided by the Technology Acceptance Model.

**Results:**

A total of 21 individuals participated in the study. All participants owned a smartphone, almost all (19/21) felt comfortable using their phone, and few already owned a wearable (6/21). Almost all participants wore the SomnoRing for 7 nights and found it comfortable. The following four themes emerged from qualitative data: (1) the SomnoRing was easy to use compared to other wearable devices or traditional home sleep testing alternatives, such as the standard polysomnogram technology for sleep studies; (2) the patient’s context and environment, such as family and peer influence, housing status, access to insurance, and device cost affected the overall acceptance of the SomnoRing; (3) clinical champions motivated use in supporting effective onboarding, interpretation of data, and, ongoing technical support; and (4) participants desired more assistance and information to best interpret their own sleep data summarized in the companion app.

**Conclusions:**

Racially, ethnically, and socioeconomically diverse patients with sleep disorders perceived a wearable as useful and acceptable for sleep health. Participants also uncovered external barriers related to the perceived usefulness of the technology, such as housing status, insurance coverage, and clinical support. Future studies should further examine how to best address these barriers so that wearables, such as the SomnoRing, can be successfully implemented in the safety net health setting.

## Introduction

Poor sleep health is increasingly recognized as a significant public health problem. It has been associated with a multitude of negative health problems including higher hospitalizations and poorer health outcomes, such as cardiovascular disease, obesity, and mental health, as well as a significant cost to the health care system (estimated US $100 million per year) [1].

Disparities in sleep health among minoritized communities as well as individuals with low socioeconomic status have been documented [2-4]. For example, racial or ethnic minorities have more adverse outcomes on multiple dimensions of sleep health including shorter sleep durations, less deep sleep, inconsistent sleep timing, and lower sleep continuity in comparison to Whites [5]. Moreover, sleep health is a contributor to health disparities—for example, a study showed that substantial proportions of racial differences in cardiometabolic risk can be explained by differences in sleep duration and sleep efficiency, respectively [6].

A growing body of evidence suggests determinants of sleep health disparities are multilevel, consisting of individual (genetic factors and psychosocial stress) as well as upstream social and contextual factors (family structure, insufficient access to care and structural discrimination, and exposure to environmental pollution) that interact to shape sleep health [7]. For example, factors affecting sleep hygiene, like consistency in sleep times, quiet, and temperature, are more difficult to achieve among individuals who share sleeping spaces with multiple family members, live in high-density housing, and lack access to air conditioning or heating [3,8]

Safety net health systems organize and provide health care to uninsured and publicly insured patients, who are disproportionately people with low income and communities of color [9]. In these resource-limited settings, safety net organizations face structural barriers including lack of subspecialty access, staffing constraints, and fragmented information technology to address patients’ sleep health [10]. Despite persistent disparities in sleep disorders, like obstructive sleep apnea, among marginalized populations [1-4], these groups are often less likely to access services that can improve sleep health and sleep management in such settings [11,12].

Digital tools, such as consumer wearable sleep technologies (eg, wristbands, smartwatches, and rings), that capture biosignals and multidimensional sleep data hold promise in clinical settings, especially in the safety net health system. They are relatively low-cost devices that facilitate real-time feedback and information sharing between patients and providers; enable monitoring and detection of patient’s sleep-wake patterns and improve treatment responses and recovery, as well as provide other potential benefits to clinic-level outcomes around staffing and cost [13-17]. Despite their potential, studies assessing the benefits and usability of wearable digital sleep technologies within and outside of clinical settings among vulnerable populations, such as Medicaid patients, are scarce [17,18]. One of the first steps toward reducing sleep health disparities is to evaluate the use and acceptance of these digital health tools within populations with low income.

The aim of this study is to explore the acceptability and usability of a wearable sleep tracking device (SomnoRing [19], also referred to as the “wearable sleep device,” below) among diverse safety net patients who have sleep problems, grounded in the Technology Acceptance Model (TAM), which has been used previously to understand the acceptance of other health care technologies [20]. Guided by the TAM, the study team qualitatively explored factors influencing the acceptance of a ring-type wearable device for sleep monitoring among English- and Spanish-speaking patients with low income seen at a safety net clinic.

## Methods

### Study Design

This study used a qualitative description approach to explore the acceptability and usability of a wearable sleep device among a sample of linguistically, racially, and ethnically diverse patients across age groups seen in a safety net clinic. The study team conducted semistructured, in-depth qualitative interviews to gain an in-depth understanding of the acceptability and usability of the wearable sleep device.

The SomnoRing device is a wearable technology for sleep monitoring in the form of a ring, and measured signals and embedded actigraphy can be used to measure pulse, continuous blood oxygen saturation, and movements. These measurements permit the calculation of oxygen desaturation index, heart rate variability, surrogates for sleep versus wake time, and sleep stages. The wearable sleep comes in small, medium, and large sizes and includes a Universal Serial Bus charging adaptor. Using a paired mobile app, users can see summarized reports of their sleep architecture, sleep stage distribution, and oxygen desaturation frequency, the latter reflecting possible sleep breathing disorders, such as obstructive sleep apnea. The app was still in development mode during this usability study; thus, this study specifically focused on the acceptability and usability of the wearable sleep device. However, study participants did use the beta version of the app in order to share app output reports with their clinical care team during their ongoing care.

### Setting and Participants

The study included patients at a multidisciplinary group practice (pulmonary, critical care, and sleep medicine) founded in 2000 in Redwood City, California. Primary care providers refer patients to the sleep specialists in the practice, and specialists then arrange sleep studies as appropriate. The practice serves diverse patients, with approximately 30% of patients preferring a language other than English and 48% on Medicaid or uninsured.

Eligibility criteria included age of more than 18 years, English- or Spanish-speaking, and an initial evaluation of obstructed sleep apnea which is most appropriate for limited cardiopulmonary testing. Our focus was on patients with suspected sleep breathing disorders, such as obstructive sleep apnea. Patients with primary insomnia or other suspected sleep disorders were not included. If a patient was suspected to have primary insomnia or other sleep disorders, they were not eligible for the study and were usually referred for a laboratory sleep study or polysomnogram. Additionally, to be eligible, participants had to complete a home sleep test using a Food and Drug Administration–cleared type III device, the ResMed ApneaLink, for one of the nights of the SomnoRing testing [21]. Patients came into the clinic for the home sleep test as part of the routine care and were then recruited from that pool to participate in testing the SomnoRing. The study excluded patients who were pregnant, with limited ability to walk due to a disability or medical condition, or experiencing active psychosis or mania.

### Recruitment

Recruitment and screening took place between February 2021 and December 2021. Clinic staff recruited patients for the study by approaching eligible patients in the clinic or calling them over the phone. Clinic staff also distributed flyers to patients and placed advertisements in the clinic waiting rooms. The study team employed purposive sampling with the aim of recruiting participants that represented a diversity of perspectives, roughly equal English and Spanish speakers, and spanning a range of age groups. The clinic staff and the research team employed purposive sampling by conducting weekly meetings to evaluate existing participants recruited for the study and identify gaps in achieving the previously mentioned aims. This helped the clinic staff strategize which patients to invite to the study based on upcoming appointments with the clinic. Clinic staff explained to potential participants the scope of the study, including wearing the SomnoRing for 7 days and completing an interview over the phone with a research staff member. After individuals consented to participate, clinic staff gave participants the wearable sleep device to use for at least 7 continuous days with additional instructions (ie, charging the device, downloading the companion app, and returning the device to the clinic after at-home use). Although the wearable sleep device has a companion app, participants were not expected or required to engage with it other than taking screenshots of output reports and metrics to send to clinic staff through email or text message. Clinic staff advised participants to use the wearable sleep device for at least 7 days and explained that after 7 days the study team would contact participants to invite them to a qualitative interview. The research team contacted participants through phone 7 days after the patient received the wearable sleep device to assess interest in the interview. The study team attempted to contact each participant 5 times. All participants provided written consent and completed the interview remotely at a time of their choice.

### In-depth Interview

The study team conducted in-depth, individual interviews over phone or Zoom using a semistructured interview guide. The open-ended, semistructured interview questions assessed participant motivations for seeking out sleep care, current sleep care experience, device prior experiences with health technology, perceptions, motivators, and barriers to using health technology, expectations from health technology platforms and wearable devices, as well as feedback focused on the usability and usefulness of the SomnoRing technology with respect to its integration into a sleep study (see interview guide in Multimedia Appendix 1). At the end of the interview, the study team conducted a 10-item validated usability questionnaire, the System Usability Scale [22], along with optional demographic questions including gender, race or ethnicity, age, preferred language, as well as questions about medical coverage, education, and digital literacy. A team member conducted the interview while others took notes (LP, MS, and KN). Each interview lasted approximately 60 minutes and was audio recorded. Interviews were professionally transcribed and, if conducted in Spanish, translated. Each participant received a US $25 gift card incentive for their time.

### Analysis

The study team used a combination of inductive and deductive analytic approaches. First, the team used a qualitative, inductive thematic analysis approach to organize interview codes into themes and subthemes to reflect the participants’ experiences without a priori hypotheses [23]. Under the guidance of the senior author (KHN), at least two trained researchers (led by LP and MS) independently read and coded transcripts to capture initial significant and relevant experiences and concepts related to the focus of the study. The coding team then discussed and reconciled discrepancies in the initial codes. KHN and LP refined the codes to create a final codebook which LP used to code the remaining transcripts. After completion of the coding, LP and KHN interpreted the codes and identified initial subthemes based on the relationships of the codes. Codes were then deductively mapped into key model constructs of the TAM. In TAM, a user’s adoption and acceptance of technology are influenced by two of the following primary factors: (1) perceived usefulness defined as “the degree to which a person believes that using a particular system would enhance his or her performance” and (2) perceived ease of use defined as “the degree to which a person believes that using a particular system would be free of effort” [20]. Furthermore, perceptions of usefulness and ease of use are mediated by external variables including individual differences, system characteristics, social influences, and facilitating conditions [24]. The research team analyzed interview codes using a constant comparison method across interviews to identify patterns and themes. The first author synthesized and refined the main themes and then identified relevant quotes and excerpts as supporting data. The team organized and coded transcripts using Dedoose, a qualitative analysis software. The exploratory analysis also considered differences between English- and Spanish-speaking participants within the final themes.

### Ethics Approval

This study was approved by the University of California San Francisco Institutional Review Board (20-30579), and all participants provided verbal consent to participate in the in-depth interviews. Participants were given the opportunity to ask questions before deciding to participate and were told that they can discontinue the study at any time with no repercussions. All participants were compensated with a US $25 gift card.

Patient identifiers were unlinked from study data, and we stored interview recordings on a secure network drive while they were needed for the study. Transcripts of the recordings were deidentified before analysis and identified versions will be destroyed 3 years after the study is completed.

## Results

### Overview

All participants (n=21) consented to wear the SomnoRing for 7 days and participate in an interview with the research team (Table 1). About half were English speaking (n=12) and the rest were Spanish speaking (n=10). Of the 21 participants, 15 (68%) reported wearing the SomnoRing continuously for 7 days during the study period.

Despite the wearable sleep device’s written instructions and app only being available in English, both English- and Spanish-speaking participants were pleased with the wearable sleep device’s ease of use, as demonstrated by the mean System Usability Scale score of 88 out of 100 (Table 2), implying that the wearable sleep device’s characteristics are compatible with widespread use.

**Table 1:** Participant characteristics.

		Spanish, n (%)	English, n (%)
**Gender**			
	Male	5 (24)	6 (27.5)
	Female	4 (19)	5 (24)
	Prefer not to say	0 (0)	1 (4.5)
**Age** **(years)**			
	26-35	5 (24)	5 (24)
	36-45	2 (10)	3 (14)
	46-55	1 (4.5)	4 (19)
	≥56	1 (4.5)	0 (0)
**Race or ethnicity**			
	White/not Hispanic	1 (4.5)	8 (38)
	Hispanic	8 (38)	1 (4.5)
	Native American/Pacific Islander/other	0 (0)	3 (14)
**Education**			
	Did not graduate high school	2 (9)	0 (0)
	High school/general educational development	4 (19)	4 (19)
	Attended some college	2 (10)	1 (4.5)
	Graduated college	1 (4.5)	5 (24)
	Obtained graduate degree	0 (0)	2 (10)
**Mobile or digital literacy**			
	Very comfortable	7 (33.5)	7 (33.5)
	Comfortable	2 (10)	3 (14)
	Somewhat comfortable	0 (0)	2 (10)
	Not comfortable at all	0 (0)	0 (0)

**Table 2:** System usability scale scores: total, by gender, and language.

		Participants, n	Value, mean (SD)
Total		21	88.1 (9.8)
**Gender**			
	Male	12	87.5 (9.6)
	Female	9	88.1 (10.2)
**Language**			
	English	12	87.1 (9.7)
	Spanish	9	88.2 (10.8)

### Overview of Qualitative Findings

In the in-depth interviews, the study team identified four overarching themes emerging from participants’ experience with the SomnoRing (Table S1 in Multimedia Appendix 2).

#### Theme 1: Participants Perceived the Wearable Sleep Device Had Greater Ease of Use Compared to Other Consumer Wearables or Prescribed Sleep Devices

Overall, participants felt that the wearable sleep device was comfortable and easy to use with minimal difficulty wearing it each night for the study. Participants mentioned the device’s simplicity, minimalist design, and lack of intrusion as contributing to ease of use.

First, most participants already had prior knowledge or ownership of wearables and compared SomnoRing’s ease of use to other wearables they had used. In general, participants preferred using the SomnoRing versus other digital devices for supporting their sleep health. Six participants previously or currently owned wearables (such as smartwatches or activity trackers) and reported that the wearable sleep device was simpler and easier to use. For example, 1 participant felt that, unlike the other devices, the SomnoRing was specifically for sleep, and she felt “the simpler the better.” Another participant remarked that the minimalist design of the SomnoRing made it appealing aesthetically and comfortable and that it was not as bulky as their other wearable device that left “imprints” on their wrists.

Overall, participants found the wearable sleep device comfortable to wear and hardly noticeable to the extent that it did not disrupt their sleep. This comfort was often noted in contrast to the other traditional home sleep testing devices provided by the clinic for other remote sleep assessments. One participant described the traditional device as, “kind of obtrusive, very uncomfortable to sleep with” whereas they didn’t “even know [the wearable sleep device was] there.” Another participant with a physical disability felt that the wearable sleep device was more comfortable, less cumbersome, and less complicated to use than other sleep devices he had used. This participant’s experience with the wearable sleep device was more straightforward and integrated with his sleep routine as it was easy enough to “put it around right before [he went to bed] and take it off as soon as [he] woke up.”

In addition to the simplicity of the SomnoRing, participants felt that the wearable sleep device was more accurate in capturing their quality of sleep because it caused minimal interruption to their sleep routine. A participant shared that they felt the wearable sleep device provided a “more accurate picture than the other sleep studies” because the straps and tubes from the traditional sleep test caused “a difficult time sleeping…being annoyed by the shape [of the device]” as well as a “paranoia” of accidentally unplugging the machine while they slept. In contrast, they felt confident to wear the ring through the night without any disturbance to the data because it had “no moving parts….so [they didn’t] have to worry about it.”

Participants also provided constructive feedback about how to further improve the ease of use of the SomnoRing, such as by improving the fit and battery. For the battery, participants shared “the hardest part was probably plugging it in to charge it” because they often found it difficult to remember to do so. One participant reflected on how they went “straight to work, and [they] didn’t charge the ring” because of the rush of the day. By forgetting to charge the ring when they came home, they found that “there [wasn’t] enough battery in there for the night,” which either made them wear the SomnoRing an extra night or delay their sleep to wait until the SomnoRing battery was charged. As for fit, the wearable sleep device was either too snug for some participants as it got “a bit uncomfortable by the end of the night” since “it leaves you with a kind of feeling like when you wear really tight socks.” A few others were concerned that the wearable sleep device would not securely stay on their finger.

#### Theme 2: Participants’ External Environment or Context Influenced Overall Acceptance or Intention to Use the Technology

Overall, family and peer influence served as key motivators for participants to seek help with their sleep problems, and more specific to the wearable use, participants’ housing status, access to insurance, and expense of devices were discussed as more direct barriers to using the SomnoRing in everyday life.

Many participants’ motivations to seek help for their sleep problem and, subsequently, to adhere to a wearable like the SomnoRing, were rooted in a desire to improve their sleep problem for their family’s benefit. Participants often shared that they sought help for their sleep because of a partner’s concern, especially when their “snoring [interrupted their partner’s] sleep.” Another participant reflected on his nephew’s influence on him to try a continuous positive airway pressure machine as it “really woke [him] up to [him] not just being a loner here, [but actually] there’s a lot of people using them.”

However, despite encouragement from loved ones, participants commented on costs and insurance status inhibiting their prioritization of sleep care and ability to afford technologies like the SomnoRing. One participant sensed they were experiencing sleep problems and wanted to “figure it out,” but was deterred from seeking help because they “didn’t have insurance for a long time.” However, they commented that the wearable sleep device prompted them to prioritize their sleep health. Furthermore, participants shared that the cost of some wearable devices could be prohibitive. One said, “it’s a waste…they [other devices] cost US $400 or US $300.” Others said that, although they would “like one of those [smart]watches,” their partners “don’t like [them] spending money.” Additionally, participants’ housing situations could be a barrier to the use of wearables like the SomnoRing. One participant said she found it difficult to charge the ring as she “would have to be careful [because she] moved around a lot” between homes.

#### Theme 3: The Clinic Staff Played Critical Role in Participants’ Interest in the Device and Its Perceived Usefulness to Their Sleep Management

The clinical care team influenced participants’ acceptance and intentions to use the technology. During the initial onboarding into the study, clinic staff spent time understanding participants’ sleep histories. For example, participants commented that the nurse served as emotional support and established a foundation of trust for participants to listen to care recommendations. Another participant felt compelled to ensure they fully completed the study and even “wore it an extra night…just to be safe and make sure that there was enough data for [the nurse] to really look at it.”

Clinic staff also played a key role in onboarding participants to use the wearable sleep device. Once participants consented to participate in the study, the clinic’s nurse ensured that all participants successfully downloaded the app, connected to the SomnoRing and app, and could follow the user flow to ensure data was captured and tracked—which directly impacted ease of use. Most participants cited they did not have to use instructions because the nurse practitioner “pretty much showed [them] how to do it, and it wasn’t a problem,” making participants feel confident in using the SomnoRing. Another participant said, “having someone walk [them] through the steps was helpful.” Participants also valued ongoing technical support during and after the study, such as clinical staff making themselves available to support participant inquiries over text message or call during the study. When the study reached completion, clinic staff also helped participants interpret their data and “go over all the results.” Although data interpretation of the accompanying app was out of scope in this study, participants commented that understanding how to improve their sleep quality and routine impacted the usefulness of the SomnoRing. Participants “looked forward…what [the clinical team has] to suggest” as they were curious about how to make changes to their sleep and trusted the nurse’s recommendations.

#### Theme 4: Despite the App Not Being Considered Central to the Study, Participants Still Had an Interest in Understanding the Complex Data and Metrics in the App and Needed More Assistance With Data Interpretation and Related Behavior Changes

Although participants were only required to share screenshots of app output data with the clinical team for care purposes, it was clear that patients were interested in using the app to engage with their data. All participants commented on their interest in “wanting to know more” about their sleep health through the app and saw the value of the metrics presented within it. One participant said they were “enjoying so much watching it on a daily basis and seeing how different every single night was.”

In reflection of their voluntary app usage, beyond just “watching” the app metrics, participants said acronyms or clinical jargon made it difficult to understand indications of good or poor sleep health. Participants were unfamiliar with acronyms like “REM” (rapid eye movement) and “PR” (pulse rate). Despite being able to click on a “?” icon that provided definitions of such terminology, participants still communicated a need for additional context to understand the meanings of these terms. Furthermore, the app’s daily sleep report reflected the participant’s averages and percentages for each sleep metric, but many expressed that it did not clearly explain whether that meant their sleep quality was poor, on track, or exceptional.

Beyond additional interpretation of data, many felt like the app or SomnoRing should be clearer about specific and actionable behavior changes that might improve sleep quality. As it was currently presented, participants didn’t feel inclined to “look into [the reports] that deep to actually try to change anything.” A majority of participants cited that they did not change anything about their sleep routine, but rather, “took those results [from the app] and sent them” to the clinical team for interpretation. Participants often associated the wearable sleep device and app together when assessing perceived usefulness. Since participants did not always understand the app metrics and reports, this affected how they perceived the ring’s usefulness. For example, a participant said the ring just “gives you information, it is not an aid.”

### Exploratory Analysis by Language

Finally, in secondary analysis, the study team also compared the main themes across English versus Spanish speakers. Overall, the team found minimal difference in how participants perceived the wearable sleep device across language groups. However, some Spanish-speaking participants experienced friction because they wanted to translate the content and metrics displayed in the app from English to Spanish. One participant said that they would take “a screenshot of whatever [they] needed and then [go] to Google Translate, Chrome, copy and paste it and then the translation came up.” Particularly for these participants, the clinical support team was even more essential for translation and interpretation of results, to teach them “what everything means, how it’s calculated, why it goes up or down.”

## Discussion

### Principal Results

This study examined people with a diagnosed sleep disorder using the TAM as a framework. In general, the study team’s findings suggest that the SomnoRing was perceived to have high ease of use as a sleep-tracking device with minimal interruption to sleep patterns. Participants perceived the SomnoRing to be more comfortable and easier to use than other wearables or sleep devices. Many participants, both English- and Spanish-speaking, noted that perceived ease of use and usefulness were highly influenced by external variables, such as family and peer influence, housing status, insurance status, and device costs—which are critical for considering the uptake and spread of these technologies to reach diverse users. Furthermore, the clinical staff played a central role in providing both clinical and technical support surrounding the device. Last, we found that participants were interested in the app to understand their sleep data. However, since the app was not the focus of this study, some participants had difficulties interpreting their sleep data. This finding highlights the importance of combining technology with the interpretation or actionable steps, such as better definitions in layman’s terms, ranges to help interpret healthy or unhealthy levels, and recommendations on how to act upon the data to change their behavior and, in turn, sleep better. Developers of connected medical devices need to attend to software as well as hardware, and in particular, incorporate principles of universal design that make apps and wearables accessible to a broad range of populations [25]. In addition, our findings suggest a potential interaction between our TAM-mapped themes. For example, the perceived usefulness of the device was directly impacted by the established relationships between patients and the clinical team, and these intersections between TAM domains should be explored in future research.

### Comparison to Previous Work

This study is similar to other studies examining the usability of sleep wearables, such as an emphasis on comfort and minimal sleep disruptions and the need to link sleep data with actionable recommendations for behavior change [26,27]. However, this is the first study to our knowledge that studied the acceptability of a sleep wearable directly linked to care provided in a safety net clinic. This is critical given the significant portion of our findings that highlighted the need for clinical staff recommendation and reinforcement of technology use for the participants in this setting.

### Limitations

Key limitations of the study include gaps in data collection and participants’ lack of clarity on the app’s role in the study. Specifically, the research team could have collected data on the amount of time clinic staff spent with patients to better understand technical support needs and how to integrate such technology into clinical workflows. Furthermore, the clinic adapted sleep clinic practices to the COVID-19 pandemic, carefully screening patients for active symptoms; thus, acutely ill patients may have been excluded from the study. However, we do not anticipate this impacting our usability findings, given that all included participants were actively being treated for their ongoing sleep conditions. Finally, given that the SomnoRing app was not ready for study inclusion, it was not emphasized as part of the study protocol. However, because patients had to use the app to begin and end the SomnoRing monitoring periods, they took interest in the app data and metrics, which in turn, influenced their overall study experience and feedback.

In safety net settings, the treatment of sleep disorders can be especially challenging. Safety net health systems struggle with limited resources for staffing and fragmented information technology [11,12]. Our study underscores how patient-facing technology like remote monitoring devices or wearables and smartphone-based apps can be delivered at the point of care, reaching patients when and where they need it. Embedding technology successfully in health care requires evaluation of the technology in the context of real-world practice within the health system [28]. To understand if technology can improve sleep treatment across patient groups and settings [13], approaches must be tested in real-world settings like the safety net and among patients who face barriers to technology access and use [29-35].

### Conclusions

There is potential for wearable sleep devices to positively impact sleep health among safety net populations. The participants in our study perceived SomnoRing to have a high ease of use. However, this study highlighted the importance of external variables, such as housing status, insurance coverage, and clinician support for understanding how safety net populations engage with such technologies. The TAM can provide a useful framework to examine how these technologies are accepted among target groups, like safety net populations. Moving forward, there is a need for more research on the usability and context of use of wearable devices, especially within the context of real-world clinical practice, to inform how to integrate these technologies into ongoing sleep intervention models and care operations.

## Appendix

Multimedia Appendix 1 Interview guide.

Multimedia Appendix 2 Supplementary table.

## Abbreviations

TAM: Technology Acceptance Model

## Data Availability

The codebook for the qualitative analysis in this study is available from the principal investigators (CRL or US) upon reasonable request.
