# Supplementary material for: Acceptability and Usability of a Wearable Device for Sleep Health Among English- and Spanish-Speaking Patients in a Safety Net Clinic: Qualitative Analysis
Source: JMIR Form Res. 2023 Jun 5;7:e43067. doi: 10.2196/43067 (PMC10280334; doi:10.2196/43067)
Supplement: Multimedia Appendix 1 [file formative_v7i1e43067_app1.docx]

**Interview Guide**

*Section details are in italics and will not be read aloud during the interview.*

***Overview***

1. *Interviewee’s background: two survey questions about general health and health/disease conditions.*
2. *Technology use: five survey questions about interviewee’s technology and internet use.*

***Interview Guide*** I am going to move onto the interview now. At the end, I’ll have a couple of more questions like the section we just finished. Now I’m going to ask you questions about your experience with your sleep and about the wearable sleep device. This interview will be audio recorded. Is that ok with you?

*If yes, begin recording and continue below.*

*If no, then explain that the study cannot continue and thank them for their time. [End call]*

Alright, I am now recording the conversation.

Thank you for being part of this study. We are interested to learn more about sleep and your experience with the wearable sleep device. There are no right or wrong answers, and we will not share your name or any identifying information as part of this study. You may skip or decline to answer any interview questions I ask today. You can also let me know if you would like to stop being part of this study. Do you have any questions for me at this time?

*Answer any questions the participant has (see* [*Q&A*](about:blank) *above, if applicable). In there are no questions move on to interview.*

Feel free at any time to ask me to repeat a question or ask it in a different way if it doesn’t make sense.

***Section 1 – Introduction.*** *The purpose of this section is to help the participant become comfortable with the interview process and to introduce a few broad questions about sleep and how they arrived at seeking care for their sleep problems. Duration: 5 minutes.*

To start: Problems with sleep are common. By the time patients go to a sleep clinic they may have tried different ways to manage their sleep or tried treatment to help with their sleep issues.

1. What first brought you to the sleep clinic?
   1. Probes: When did you first notice a sleep problem? How does sleep affect your day-to-day activities? How does it affect your work and family life?

***Section 2 – Sleep Management.*** *The purpose of this section is to further explore what participants have done to manage their sleep health or sleep problems, whether in or outside of the clinic. We would like to understand the extent of participants’ experiences, including challenges and things that worked well, in obtaining care or seeking solutions for their sleep issues. Duration: 20-25 minutes.*

In this next part, I want to hear about the things you’ve done to help fix some of the sleep issues you have been experiencing.

1. Tell me how your doctors learned about your sleep challenges
   1. Probes: most doctors start by asking questions about sleep, and then doing a sleep study, at home or at a clinic. Have you had a sleep study? At home or at a sleep clinic? Have you ever used a CPAP machine or at home device, etc., to treat your problems with sleep? Did you receive a diagnosis?
2. Tell me about what your sleep doctor has recommended to help with your sleep.
   1. Probe: For each treatment, ask: When did you first try that? Was that helpful? What did you like? What didn’t you like?
   2. Probe: any changes to your diet, exercise, or habits, like meditation, to improve sleep? Any sleep scheduling like sleep restriction? Any sleep training or therapy for sleep?
3. Did you ever try tracking your sleep with an app on your phone or a device like a FitBit? *Participant may report tech-tracking solution (i.e., sleep tracking app or other device like a Fitbit, OuraRing, etc).* Tell me more about [NAME OF PRODUCT] you’ve tried. When did you first start using it? Was it helpful? What did you like? What didn’t you like? Would you use it again? Why or why not?
4. What are the most important things you do to manage your sleep? Has this changed over time?

***Section 3 – Experience with the wearable sleep device.*** *The purpose of this section is to assess participants’ specific experience with the wearable sleep device used in this study. We would like to learn more about the usability of the device, benefits, and any challenges with the wearable sleep device. Additionally, we would like to compare the usability of the* wearable sleep device *to other therapeutics/treatments/testing (e.g., CPAP) that they may have done at the clinic or outside the clinic. Duration: 20-25 minutes.*

Thank you for sharing your experiences with sleep so far. In this study, you wore the wearable sleep device, a device to track your sleep, for seven days. In the next several questions, I will ask more about the wearable sleep device to learn about what the experience was like for you.

1. Thinking about your week with the device, what are your thoughts about the wearable sleep device?
   1. How many nights did you wear the device? If there were nights that you did not wear the sleep device, can you tell me more about what prevented you from wearing it?
   2. Tell me more about using the wearable sleep device. Were the instructions that came with the wearable sleep device clear? Did you have any trouble using the wearable sleep device? How did wearing the device fit in to your daily routine? What was the hardest part about using the wearable sleep device? What would you change?
2. Tell me how you used the wearable sleep device app.
   1. *Participants will vary in how they used the app.* What motivated you to use the app? Was it easy to find and download? What do you see or look for when you’re in the app? How often did you use it? What are some tasks that you used the app for? What do you like about the app? What don’t you like about it? What would you change? Was it helpful? Would you continue to use it? Why or why not? Would you recommend it to a friend? Did you change your behaviors based on what you saw in the app?
   2. *Participants might say that they did not use the app.* Tell me about why you don’t use the app? What don’t you like about it? What might motivate you to use the app? Do you think the app is useful? If not, what might make it useful for you? What do you think the wearable sleep device/data is going to do and help them with?
   3. *Participant might say that they have not heard of the app before.* Has your sleep doctor ever mentioned the app? Have you heard about the app before? Would you be interested in using an app to help with your sleep? Why or why not?
3. How does the wearable sleep device compare to other things you’ve done or tried to help with your sleep?
   1. Do/did you use another test or device at home to track your sleep? Was using the wearable sleep device easier or more difficult than other things you’ve tried? How so?
4. Tell me your thoughts about using the wearable sleep device in the future.
   1. How would it be helpful? Would you recommend it to a friend?
5. How did your experience with the wearable sleep device change your thoughts about using devices or other digital tools, like apps, to help with your sleep?
6. Next, I will read a few statements about both the wearable sleep device and the app. On a scale of 1-5, 1 being strongly disagree and 5 being strongly agree, please indicate how you feel about each of the following statements:

Response options for all questions:

1. Strongly Disagree
2. Disagree
3. Neither agree nor disagree
4. Agree
5. Strongly agree
   1. I would like to use the wearable sleep device frequently
   2. The wearable sleep device is unnecessarily complex.
      1. *Clarifications: Not simple, difficult to understand how to use*
   3. The wearable sleep device was easy to use.
   4. I would need support from a technical person to be able to use the wearable sleep device.
   5. The different functions in the wearable sleep device were well integrated.
      1. *Clarifications: The wearable sleep device, the app, and the experience went smoothly and were well connected*
   6. The wearable sleep device was too inconsistent.
      1. *Clarifications: Data measurements, charging, and/or other functionality and were not reliable*
   7. Most people could learn to use the wearable sleep device very quickly.
   8. I found the wearable sleep device very cumbersome (awkward to use).
   9. I felt very confident using the wearable sleep device.
   10. I needed to learn a lot of things before I could get going with the wearable sleep device.

*Conclusion:* Is there anything else you would like to share about your wearable sleep device experience or overall experience with sleep care?
