# Supplementary material for: Acceptability and Usability of a Wearable Device for Sleep Health Among English- and Spanish-Speaking Patients in a Safety Net Clinic: Qualitative Analysis
Source: JMIR Form Res. 2023 Jun 5;7:e43067. doi: 10.2196/43067 (PMC10280334; doi:10.2196/43067)
Supplement: Multimedia Appendix 2 [file formative_v7i1e43067_app2.docx]

Supplementary Table 1. Participant quotes organized by theme

| **Theme** | **Exemplary quotes** |
| --- | --- |
| Theme 1: Participants perceived the SomnoRing→ had greater ease of use compared to other consumer wearables or prescribed sleep devices | “It’s not trying to be anything more than what it is so I don’t – if it was more like a Fitbit, Apple Watch or whatever, I can imagine that there might be more to look at and to explore, but if it’s trying to be for sleep study, then I think the directness is better. I’m just imagining all the populations I’ve worked with and especially aging population, the simpler the better.”  “The Fitbit, I have a bulkier kind of one, so it leaves a lot of imprints in my wrist a lot of times. The ring would be so much better in place of that.”  “Since I'm in a wheelchair, it’s hard for me to put [strength] around my chest and make sure everything is right, take things on. I don’t normally sit on the edge of a bed and [Unintelligible] to lie down. It’s a little more complicated for me. It was a bit challenging…[with the FitBit] I stopped wearing it is because being on a wheelchair, I didn’t like the feeling of something around my wrist…for the four nights I wore [the ring] I would put it around right before I went to bed and take it off as soon as I woke up.”  “The other sleep study was kind of obtrusive, very uncomfortable to sleep with, and the ring, I don’t even know it’s there. In a sense, it kind of at least in my situation probably has a more accurate picture than the other sleep studies just because I had such a difficult time sleeping with the strap and the tubes in my nose and being annoyed by the shape of it. Almost like kind of paranoia of like one of the tubes got unplugged from the machine in that sleep study, I messed up. It’s like no moving parts to the ring and I know it will work so I don’t have to worry about it.”  "The hardest part was probably plugging it in to charge it. I mean, really it was nothing to do, or just remembering to put it on. I mean, there’s really nothing hard about it at all." |
| Theme 2: Participants’ context influenced overall acceptance and/or intention to use the technology | "She started to realize that when I’m asleep, I go through periods of maybe a few seconds where I don’t seem to breathe. So, she started to be a bit worried about me, and she said, ‘why don’t you go get checked by a doctor to see if you’ve got some kind of problem?’...So, it was also about that, the fact that with my snoring I interrupt her sleep, and with the baby and everything - and that was the main reason why I went to get myself checked."  “I’m retired now and it’s funny that you mentioned that because I was a firefighter and my nephew is a firefighter also…he says, ‘You know, uncle, a lot of guys are sleeping with these CPAP machines at the firehouse.’ Now he says it’s more common than you think. He says out of a group of seven or eight, three or four of them might have the machines…That really woke me up to I’m not just a loner here, there’s a lot of people using them.”  “I just think – I presumed that I was getting less sleep, but even then, like aside from that, aside from the past six months, I – there were still sleep patterns that I was interested in and would’ve been desirous of figuring out. Yes, and I didn’t have insurance for a long time and so I’ve been – I have insurance. I can take care of myself now.”  "I suffer from gout and sometimes my feet hurt, and I can’t get out for a walk...one time I wore the ring out walking to monitor my steps and everything, and I liked it because it showed me, I’d used 400 calories and done 8000 steps...I liked it. But watches and stuff, no I have never had one. I’d like one of those watches, but my wife doesn’t like us spending money."  "I don’t have an Apple Watch because it’s too fancy for me. At work, I don’t have to wear a watch if I don’t want to. It’s a waste. They cost 400 or 300 dollars. I have friends who have them and they say they work well.”  “I’d have to be very careful…I moved around a lot and so I was very conscious of it until it was charged and then I put it away ready for the evening…I was in an RV [recreational vehicle] and multiple people living in RV, moving around. When we walk around, [the ring] moves. I can imagine if I had a bathroom, I can go in and brush my teeth or something. I use the toilet in the morning and ‘Oh, there’s my charge station.’ That’s a big experience which I didn’t have.” |
| Theme 3: The nurse practitioner played critical role in participants’ interest in the device and its perceived usefulness to their sleep management | “At first, I was really nervous about talking with someone because I know that I was warned ahead of time that the questions might get a little bit more personal and intimate, and having to go with my medical history and whatnot…I’ve had some things happened to me that it’s really difficult for me to talk about, but having talked to her, she seemed to relate in a lot of ways. The questions that she asked me when I elaborated for her, it seemed like she was really listening to me. There were a few times that she actually threw in her own personal experiences…She thanked me for being so open about it and she kind of congratulated me in a way for being able to talk about it and not break down or freak out. I didn’t realize that people can get so emotional about their sleep issues, but she was extremely nice, and it was a quick visit.”  “I was supposed to wear it for seven nights. I think I wore it an extra night because I think one of the nights that I wore it, that’s when I learned how the charging worked, but I think it died on me part of the night, so I didn’t charge right. I did an extra night just to be safe and make sure that there was enough data for [the nurse] to really look at.”  “When I was speaking to [the nurse], just kind of going through my life story and the kind of different chapters I’ve had and the various traumas essentially that I’ve had… just saying like all these things are possible contributors to sleep habits. So, even though I’ve been grinding my teeth since a child, she just wanted to do a sleep study and suggested I do the sleep study for my own benefit as well to kind of see what’s happening during that time I’m trying to rest.”  “...there were instructions that came with it, but [the nurse] explained everything to me, helped me download the app on my phone and get it set-up and shown me how to work it, that it was pretty straightforward. It wasn’t hard to figure out at all.”  “The person who gave it to me at the sleep study pretty much gave me all the instructions so I didn’t read up for anything. She explained very thoroughly for about 30 minutes.”  "Sometimes I didn’t understand what the ring was saying. But the doctor told me about the range in which you should sleep, so I knew roughly whether I was sleeping or not, and whether it’s doing my body any good. I liked seeing what the ring... It was very practical. The doctor explained to me about the wake-up data, [and the light that measures charge], it was all good."  “I think now seeing how much time I spend in a deep sleep versus that light sleep kind of makes me realize that I think I need to find a way to change it so that I can get into that deeper sleep and maybe that would help me, but I’m looking forward to talking to [the nurse] and going over all of the results and see what she’s got to suggest for me and what she’s able to get from the data that I provided for them.” |
| Theme 4: Despite the app not being considered as central to the study, participants still had an interest in understanding the complex data and metrics in the app and needed more assistance with data interpretation and related behavior changes | “Because the sleep study itself was only just the one night, I was really getting curious…I was like starting to get questions and wanting to know more, and so I went into the app to see if it told me anything about my oxygen or my pulse or anything like that, and when I went on, I was just blown away by the little meter that it gives you…I was enjoying so much watching it on a daily basis and how different every single night was. I mean, no two nights were the same. There were some that I spiked in my pulse, and then there were nights where my oxygen was a little different. It kind of made me think about, okay, was I dreaming at that point? It just put a lot of thought into my head.”  "There are abbreviations for REM which is I don’t know what that is. Then it as a light sleep and deep sleep study. It’s pretty interesting to understand the next day. I just don’t know what the REM stands for."  “I think it would be good that inside the application there is a description of that every subject means. There are some people who are ignorant, we are not doctors. So, to write what PR means, what OUDI means, etcetera.”  "I didn’t look into [the reports] that deep to actually try to change anything. I only tried to go to sleep sooner because I would look at how many hours I slept.”  “As soon as you take the ring off and set it up and go next to the phone, it was almost like instant. It came out with the result. It was shocking too. Then I took those results and sent them to [the clinic staff] within half an hour or so of reading it.”  "I didn’t quite understand the terms and what everything meant. I would click on the buttons next to them to try and get that little definition, but I still didn’t quite understand it. Maybe just having some - I don’t want to put it in a mean way, but maybe dumb it down for some of us because I feel like it was a lot more medical terms that I didn’t understand. I’d be having a definition that was a little more meant for somebody who doesn’t understand those medical terms would be a little bit better."  “It would be good to know what the normal range are. I don’t know, if one has between 90 and 95 degrees, that is normal, that is not a fever, right? If one passed 105, then they have a fever, as we call it here, right? Something like that. To give a little bit of indication what that normal range is for a normal person, one without any problems. Just to know how alarmed we should be, and to seek for help."  "Everything was clear. Mostly, because I translated it...I copied it, or I took a screenshot of whatever I needed and then I went to Google Translate, Chrome, I copy and pasted it and then the translation came up."  "I had questions. I can’t do exactly what my doctor does but the last two times I went she taught me what everything means, how it’s calculated, why it goes up or down. She explains it to me. Any doubts I have, I ask her. She explains it to me too.” |
